# High-performance liquid chromatography screening reveals HbS/β^+^-thalassemia double heterozygosity as a pediatric muscular dystrophy mimic

**DOI:** 10.1093/labmed/lmag019

**Published:** 2026-04-22

**Authors:** Carlos López-Medina, Isabel M Portell-Rigo, Virginia González-Iribarren, Carlos González-Oller, Alicia Sánchez-Crespo, Maria-Angustias Molina-Arrebola

**Affiliations:** Clinical Analysis Unit, Biotechnology Department, Poniente University Hospital, El Ejido, Almería, Spain; Clinical Analysis Unit, Biotechnology Department, Poniente University Hospital, El Ejido, Almería, Spain; Pediatric Department, Poniente University Hospital, El Ejido, Almería, Spain; Clinical Analysis Unit, Biotechnology Department, Poniente University Hospital, El Ejido, Almería, Spain; Haematology and Haemotherapy Department, Torrecárdenas University Hospital, Almería, Spain; Haematology and Haemotherapy Unit, Biotechnology Department, Poniente University Hospital, El Ejido, Almería, Spain

**Keywords:** sickle cell disease, β-thalassemia, HPLC, hemoglobinopathy, G6PD deficiency, pediatric, parvovirus B19

## Abstract

**Introduction:**

Compound hemoglobinopathies may present with variable clinical phenotypes, particularly when additional genetic modifiers coexist. Sickle cell hemoglobin (HbS)/β^+^-thalassemia can mimic other pediatric conditions, potentially delaying diagnosis. High-performance liquid chromatography (HPLC) is a widely accessible first-line screening tool that can facilitate identification of HbS/β^+^-thalassemia.

**Methods:**

An 8-year-old boy from Mali presented with recurrent severe lower limb pain and gait impairment, initially suggesting a neuromuscular disorder. Laboratory evaluation revealed severe microcytic hypochromic anemia (hemoglobin, 6.3 g/dL) with reticulocytosis and biochemical evidence of hemolysis, despite preserved iron stores. Creatine kinase levels were normal. Peripheral smear showed anisopoikilocytosis with target cells, basophilic stippling, and rare sickled erythrocytes.

**Results:**

The HPLC findings demonstrated HbS predominance (64.2%) with elevated fetal hemoglobin (8.1%), increased adult hemoglobin A_2_ (5.4%), and residual adult hemoglobin A (22.3%), consistent with HbS/β^+^-thalassemia. Molecular testing confirmed heterozygous HbS and β^+^-thalassemia variants, homozygous 3.7-kilobase α-globin gene deletion, and a pathogenic glucose-6-phosphate dehydrogenase A^‒^ haplotype. Positive parvovirus B19 immunoglobulin M suggested an additional acute trigger.

**Discussion:**

Routine hematologic parameters combined with HPLC screening can promptly identify complex hemoglobinopathies in clinically misleading presentations, enabling accurate diagnosis, appropriate management, and genetic counseling.

## Introduction

An 8-year-old boy born in Mali presented at the emergency department with recurrent episodes of severe pain in the lower limbs, predominantly involving the thighs and gluteal region, associated with difficulty walking and reduced mobility. According to his father, the patient had experienced 2 similar self-limited episodes in the past, each lasting approximately 1 week. There was no history of trauma, fever, bleeding, or recent infection.

Due to the presence of gait impairment and apparent proximal muscle weakness, an initial suspicion of a neuromuscular disorder, including Duchenne muscular dystrophy, was raised in the emergency setting. The patient was admitted for observation and further evaluation. On physical examination, he appeared asthenic but hemodynamically stable and afebrile. Mucous membranes were pale, with no petechiae, adenopathy, or organomegaly. Cardiopulmonary examination was unremarkable. Neurologic examination revealed no focal deficits, although weakness of the pelvic girdle was suspected, likely related to pain rather than true muscle involvement.

## Methods

### Laboratory evaluation

Initial hematologic findings revealed severe microcytic hypochromic anemia, with a hemoglobin level of 6.3 g/dL, a hematocrit level of 19.7%, and microcytosis (mean corpuscular volume, 73.2 fL), accompanied by reticulocytosis (3.9%), with an absolute reticulocyte count of 107.33 ×10³/µL and an increased immature reticulocyte fraction of 0.64, suggesting ongoing hemolysis. White blood cell and platelet counts were within normal limits.

**Figure 1:**
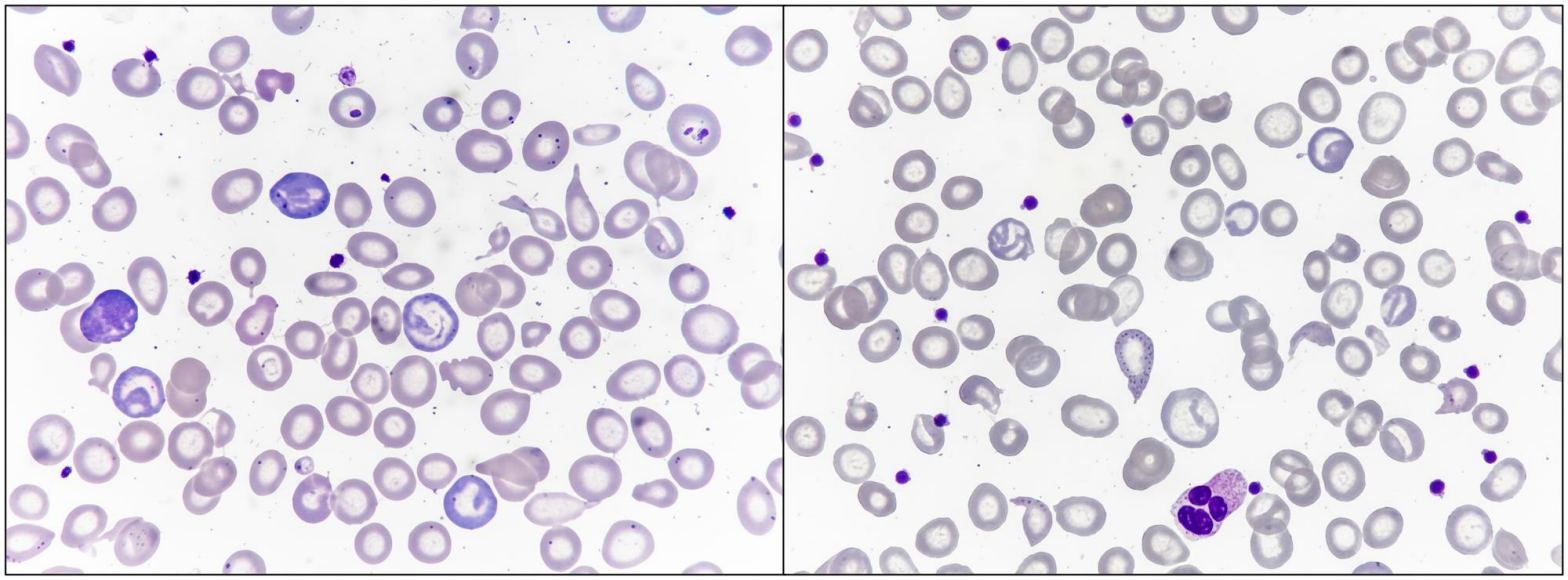
Peripheral blood smear showing microcytic hypochromic erythrocytes, anisopoikilocytosis, target cells, polychromasia, basophilic stippling, and rare sickle erythrocytes (May-Grünwald-Giemsa, ×100 original magnification).

Iron metabolism studies demonstrated preserved iron stores, with normal serum iron and transferrin saturation and mildly elevated ferritin levels (330 ng/mL), effectively ruling out iron deficiency anemia. Biochemical markers showed elevated lactate dehydrogenase (380 U/L) with normal haptoglobin levels. Creatine kinase values were normal, arguing against a primary myopathic process (Table 1). Peripheral blood smear examination revealed marked anisopoikilocytosis, with target cells, basophilic stippling, polychromasia, and rare sickled erythrocytes (Figure 1).

**Table 1:** Peripheral blood count, iron metabolism, and hemolysis markers.

Parameter	Value	Reference range	Unit
Erythrocytes	2.69	3.95-5.25	×10^6^/µL
Hemoglobin	6.3	11.8-14.6	g/dL
Hematocrit	19.7	34.0-43.5	%
Mean corpuscular volume	73.2	76-91	fL
Corpuscular mean hemoglobin	23.2	25.0-31.5	pg
Reticulocytes	3.9	1.3-2.7	%
Absolute reticulocyte count	107.33	30-105	×10³/µL
Immature reticulocyte fraction	0.64	0.30-0.54	
Serum iron	87	50-120	µg/dL
Ferritin	330	6-320	ng/mL
Transferrin	231	—	mg/dL
Transferrin saturation index	29.7	17.1-30.6	%
Haptoglobin	48	30-200	mg/dL
Lactate dehydrogenase	380	110-295	U/L
Glucose-6-phosphate dehydrogenase at 37 °C	157.5	221-570	mU/10^9^ red blood cells

Given the hemolytic anemia and the patient’s ethnic background, hemoglobinopathy was suspected. High-performance liquid chromatography (HPLC), the gold-standard technique for screening structural hemoglobinopathies and thalassemias,^1^ was performed (G11 Horiba Analyzer [Tosoh Bioscience]). A sickling test with sodium metabisulfite was positive. Capillary hemoglobin electrophoresis confirmed sickle cell hemoglobin (HbS) predominance (64.2%), with adult hemoglobin (HbA; 22.3%), elevated fetal hemoglobin (HbF; 8.1%), and HbA_2_ (5.4%) levels, consistent with sickle cell disease associated with thalassemic features (Table 2). These findings—microcytic anemia, HbS higher than 50%, elevated HbA_2_/HbF, and residual HbA—are characteristic of HbS/β-thalassemia compound heterozygosity.^2^ In addition, glucose-6-phosphate dehydrogenase (G6PD) activity was measured, revealing a decreased value of 157.5 mU/10^9^ red blood cells (at 37 °C). The hematology team concluded that the clinical presentation was more consistent with vaso-occlusive episodes related to sickle cell disease rather than a neuromuscular disorder.

**Table 2:** Hemoglobin fractions detected by HPLC and capillary hemoglobin electrophoresis.

Hemoglobin fraction	HPLC, %	Capillary electrophoresis, %	Reference range
HbA	20.6	22.3	96-98
HbA_2_	5	5.4	2.0-3.5
HbS	57.1	64.2	—
HbF	7.2	8.1	<1

Abbreviations: HbA, adult hemoglobin; HbF, fetal hemoglobin; HbS, sickle cell hemoglobin; HPLC, high-performance liquid chromatography.

Given the laboratory findings and the severity of anemia, extended molecular testing, including sequencing of the *HBB* and *G6PD* genes and polymerase chain reaction with reverse hybridization of the *HBA1* and *HBA2* genes (α-Globin StripAssay), identified the following (Table 3): a heterozygous hemoglobin S variant (NM_000518.5: c.20A>T; p. Glu7Val) and pathogenic β-thalassemia–associated mutation (NM_000518.5: c.75T>A, codon 24) in the *HBB* gene, classified as β^+^-thalassemia; a homozygous 3.7-kilobase deletion (–α³·^7^/–α³·^7^) affecting *HBA1/HBA2* in the α-globin genes, consistent with α-thalassemia trait; and a hemizygous pathogenic variant (NM_001360016.2: c.202G>A; p. Val68Met) plus a variant of uncertain significance (NM_001360016.2: c.376A>G; p. Asn126Asp) in the *G6PD* gene, corresponding to the G6PD A^‒^ haplotype associated with G6PD deficiency.

**Table 3:** Molecular genetic findings.

Gene	Variant	Zygosity	Inheritance	Classification
*HBB*	NM_000518.5: c.20A>T (p.Glu7Val)	Heterozygous	Autosomal recessive/dominant	Hemoglobin variant (HbS)
*HBB*	NM_000518.5: c.75T>A (codon 24)	Heterozygous	Autosomal recessive/dominant	Pathogenic β^+^-thalassemia
*HBA1/HBA2*	3.7-kilobase deletion (–α³·⁷/–α³·⁷)	Homozygous	Autosomal recessive	α-Thalassemia trait
*G6PD*	NM_001360016.2: c.202G>A (p.Val68Met)	Hemizygous	X-linked	Pathogenic
*G6PD*	NM_001360016.2: c.376A>G (p.Asn126Asp)	Hemizygous	X-linked	Variant of uncertain significance

Abbreviations: G6PD, glucose-6-phosphate dehydrogenase; HbS, sickle cell hemoglobin.

## Results

### Clinical course and management

Although the genetic study revealed additional pathogenic variants, the initial hemogram and HPLC hemoglobin fractions were sufficient to suspect—and molecular testing confirmed—HbS/β^+^-thalassemia compound heterozygosity, given the characteristic pattern: microcytic anemia, HbS higher than 50%, elevated HbA_2_/HbF, and residual HbA presence. In addition, serologic testing revealed positive immunoglobulin M (IgM) antibodies against parvovirus B19, which may have contributed to the increased severity of the anemia.

The coexistence of these genetic alterations explained the patient’s severe hematologic phenotype and recurrent vaso-occlusive episodes. Given his stable clinical condition, blood transfusion was not required at that time. The patient was managed conservatively with hydration and analgesia; iron supplementation was avoided. A sickle cell disease treatment protocol was initiated with hydroxyurea, folic acid, vitamin D, and specific vaccinations (pneumococcal, meningococcal, and influenza vaccines).

## Discussion

This case exemplifies how routine hemogram and HPLC—available in most clinical laboratories—can prompt suspicion of complex hemoglobinopathies, even in challenging diagnostic presentations. The patient’s initial presentation with severe pain and gait impairment, combined with proximal muscle weakness, understandably raised suspicion of neuromuscular disease (eg, Duchenne muscular dystrophy). Critical laboratory findings, however—marked microcytic hypochromic anemia, reticulocytosis, hemolytic markers with preserved iron stores, distinctive peripheral smear findings, and normal creatine kinase values—redirected the diagnostic pathway. In suspected neuromuscular disorders, laboratory parameters are critical for differentiation. Primary myopathies typically show markedly elevated creatine kinase levels and normal hematologic profiles. In contrast, our patient had normal creatine kinase, with clear evidence of hemolytic anemia and reticulocytosis, findings that effectively excluded a primary myopathic process.

The HPLC results were pathognomonic for compound S/β^+^ thalassemia heterozygosity: HbS predominance (64.2%), elevated HbF (8.1%), elevated HbA_2_ (5.4%), and residual HbA (22.3%) distinguish this condition from homozygous sickle cell disease or compound S/β0-thalassemia heterozygosity (where HbA would be absent). This case underscores the value of HPLC as a first-line screening tool accessible worldwide, particularly in resource-limited settings, where genetic testing may be delayed.

The discovery of coexisting α-thalassemia (homozygous 3.7-kilobase deletion) and G6PD deficiency (A^‒^ haplotype) further complicated the patient’s phenotype. G6PD deficiency is known to exacerbate hemolysis in sickle cell disease and increase susceptibility to acute hemolytic crises, particularly with oxidative stress or infection.^3^ The concurrent parvovirus B19 infection (positive IgM) likely triggered or worsened the acute anemia, as parvovirus B19 causes direct suppression of erythropoiesis, particularly damaging in patients with chronic hemolysis.^4^ In isolation, HbS/β^+^-thalassemia generally causes mild to moderate hemolytic anemia with variable vaso-occlusive manifestations, G6PD deficiency predisposes to oxidative hemolysis, and parvovirus B19 may trigger transient aplastic crisis in patients with chronic hemolysis. Their coexistence in this patient likely intensified the clinical severity. The presence of positive parvovirus B19 IgM and underlying G6PD deficiency were considered contributory rather than incidental, potentially amplifying both hemolysis and transient erythropoietic suppression.

The initial neuromuscular suspicion highlights key clinical discriminators between true myopathies (Duchenne muscular dystrophy, congenital myopathies), which typically present with normal hemoglobin/reticulocyte counts, an absence of hemolysis markers, and marked creatine kinase elevation, and this patient’s severe anemia with reticulocytosis and a hemolytic profile that immediately shifted diagnostic consideration toward hemolytic disorders. Early recognition of HbS/β^+^-thalassemia through HPLC is critical for preventing complications through hydroxyurea initiation to increase HbF levels and reduce HbS polymerization/vaso-occlusive episodes, infection prevention through appropriate vaccination (pneumococcal, meningococcal, *Haemophilus influenzae* type B), genetic counseling regarding autosomal recessive HbS/β-thalassemia and X-linked G6PD deficiency inheritance patterns, family screening to identify carriers and offer reproductive counseling, and ongoing monitoring of blood counts.

The coexistence of α-thalassemia may moderately ameliorate hemolysis (as α-thalassemia reduces excess globin chain imbalance), whereas G6PD deficiency is an aggravating factor that requires avoidance of oxidative triggers (medications, infections, fava beans).^5^

This case emphasizes that comprehensive hematologic and molecular evaluation is essential in pediatric patients with unexplained anemia and recurrent pain crises. This case further illustrates how careful interpretation of routine laboratory tests, rather than the use of novel methodologies, can be decisive in resolving clinically misleading presentations. Routine hemogram findings (microcytic hypochromic anemia, marked reticulocytosis, hemolytic markers) combined with HPLC screening—a gold-standard, widely accessible technique—can accurately identify complex hemoglobinopathies, preventing misdiagnosis and enabling timely, appropriate management. Recognition of coexisting genetic modifiers (α-thalassemia, G6PD deficiency) further refines clinical phenotype prediction and counseling. Early diagnosis ensures access to disease-modifying therapy (hydroxyurea, gene therapy candidates), preventive measures (vaccination), and accurate genetic counseling for families, ultimately improving long-term outcomes and quality of life.

## Data Availability

The data underlying this article are available in the article. Additional data are available from the corresponding author upon reasonable request.
